# Diagnostic whole transcriptome sequencing in a series of 1233 FFPE solid tumor samples

**DOI:** 10.1038/s41416-025-03307-8

**Published:** 2026-01-14

**Authors:** Markus Ball, Susanne Beck, Darius Wlochowitz, Tina Fuchs, Katja Lorenz, Christiane Zgorzelski, Alejandro Pallares Robles, Michael Allgäuer, Anna-Lena Volckmar, Hannah Goldschmid, Iordanis Ourailidis, Regine Brandt, Petros Christopoulos, Michael Thomas, Huriye Seker-Cin, Annette Fink, Fabian Schnecko, Olaf Neumann, Michael Menzel, Martina Kirchner, Thoas Fioretos, Peter Schirmacher, Solange Peters, Jan Budczies, Albrecht Stenzinger, Daniel Kazdal

**Affiliations:** 1 Institute of Pathology, Heidelberg University Hospital, Heidelberg, Germany; 2 Translational Lung Research Center (TLRC) Heidelberg, German Center for Lung Research (DZL), Heidelberg, Germany; 3 Department of Medical Oncology, Thorax Clinic, Heidelberg, Germany; 4 Division of Clinical Genetics, Department of Laboratory Medicine, Lund University, Lund, Sweden; 5 Department of Clinical Genetics, Pathology and Molecular Diagnostics, Office for Medical Services, Region Skåne, Lund, Sweden; 6 Oncology Department, CHUV, Lausanne University, Lausanne, Switzerland; 7 German Cancer Consortium (DKTK), Heidelberg, Germany; 8 Center for Personalized Medicine Heidelberg (ZPM), Heidelberg, Germany

**Keywords:** Predictive markers, Medical genetics, Molecular medicine, Cancer

## Abstract

**Background:**

Whole Transcriptome Sequencing (WTS) is a comprehensive alternative to targeted panels for detecting gene fusions and splice variants. To integrate WTS into clinical diagnostics, we compared its performance against established fusion assays (Archer FusionPlex and TSO500 RNA).

**Methods:**

WTS was evaluated in an initial cohort of 64 FFPE tumor samples, and quality control (QC) thresholds were defined based on missed fusions correlating with low tumor cell content (TCC < 40%). Key QC metrics included TCC ≥ 40%, RNA input ≥50 ng, ≥50 million reads, and median insert size >100 bp.

**Results:**

WTS identified 92% of known fusions in the initial cohort. Validation in 357 samples showed 100% concordance with panel-based results when QC thresholds were met. Subsequent clinical deployment across 812 diverse tumor cases detected 121 fusions, though 423 (34%) required fallback to targeted assays due to low TCC. WTS provided added value by detecting novel fusions, pathogens, and enabling oncogenic pathway analysis.

**Conclusion:**

WTS is a reliable and informative method for fusion and splice variant detection in clinical diagnostics, provided rigorous pre-analytical and sequencing QC metrics are strictly applied.

## Introduction

Ever since gene fusions were identified as oncogenic drivers in cancer they were investigated for diagnosis, prognostication, and therapy prediction [1]. For instance, the Philadelphia chromosome is known for over 50 years, resulting in the gene fusion *BCR-ABL1* in chronic myeloid leukemia [2, 3]. Since then, many different methods were developed to identify gene fusions in cancer. Methods like fluorescence in situ hybridization (FISH) and chromogenic in situ hybridization (CISH) focus primarily on the DNA level, detecting the localization of target probes to specific DNA regions which can detect translocations even with limited amounts of tumor material [4], offer a quick turnaround time and comparatively less complex instrumentation. They are widely established in clinical practice and considered for a long time as the gold standard.

RNA analyses are a reliable tool to detect fusion transcripts and to characterize the transcriptome by quantifying gene expression profiles [5]. Various molecular techniques for the analyses of the transcriptome were developed. But methods like reverse transcriptase polymerase chain reaction (RT-PCR) and microarrays allow only the interrogation of a limited number of genes. With the emergence of next generation sequencing (NGS) technologies, more extensive broad profiling of genes became possible [6, 7]. Today, a diverse range of targeted RNA-based assays is available which enable the characterization of gene expression [6, 8] and the detection of fusions in a selected set of clinically relevant fusion genes [9]. Single primer extension and hybrid capture panels work with reverse transcribed RNA, and target the biological active and oncogenic part of the later expressed fusion proteins with the flexibility of identifying unknown fusion partners [10].

Whole transcriptome sequencing (WTS), or RNA sequencing (RNA-seq), represents an even broader approach to obtain a comprehensive genome-wide view of the transcriptome by analyzing the [7] expression of all actively transcribed genes and the detection of atypical, rare, and novel fusions. Compared to targeted expression arrays, WTS offers a relatively unbiased analysis of the transcriptome and an unfocused assessment of genes fusions [11, 12].

Several approaches exist for preparing WTS libraries, including Poly(A) selection, rRNA depletion, and exome capture. Poly(A) selection enriches for coding mRNAs but excludes degraded or non-polyadenylated RNAs. rRNA depletion removes ribosomal RNA, enabling broader RNA detection, including non-coding and non-human RNA. Exome capture targets exonic regions, offering good performance with fragmented samples but limiting transcriptome-wide analysis.

Furthermore, WTS can provide additional clinically meaningful information on e.g., alternative splicing events, expression of neoantigens, differential expression analysis, single nucleotide variant detection, transcription activity of kinases or genes encoding targets for which antibody drug-conjugates (ADCs) are available [11, 13]. Along this line, an evaluation of the prevalence of different immune cell populations in the tumor microenvironment like tumor-infiltrating lymphocytes (TILs), macrophages or NK cells is technically feasible [14–16]. The latter may hold information useful for monitoring or predicting response to immunotherapy [17].

To sum it up, the use of WTS enables a comprehensive assessment of the dynamic nature of the transcriptome of a patient’s sample. Considering the vast amount of information, bioinformatic processing of the RNA sequencing data is a crucial step for an appropriate analysis, especially if WTS is translated into clinical diagnostics and guiding therapeutic decisions [18]. Hence, integrating WTS into the inevitably high-quality standards of clinical diagnostic workflows is challenging and must pass thorough technical performance characterizations [11]. The implementation of WTS in routine diagnostics has already been reported for acute lymphoblastic leukemia [19] and pediatric cancer [20].

**Fig. 1:**
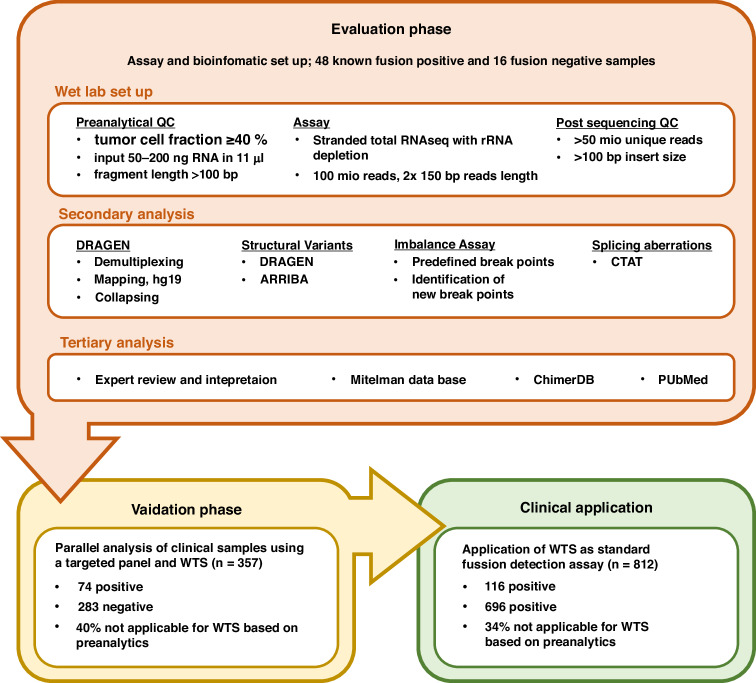
Study design and overview of the three phases of implementation of the WTS depletion assay in clinical practice. Evaluation phase shows QC parameters, methods and computational resources used for analysis. Validation phase and clinical application summarizes the experiments and application progress in this study.

In this study, we compare the performance of WTS and two targeted RNA-based next generation sequencing assays in a routine clinical setting using FFPE tumor samples covering a wide range of solid cancer types and describe the implementation of WTS into routine diagnostics in our department (Fig. 1).

## Material and methods

### Samples

All 1233 carcinoma samples included in this study were diagnosed and processed at the Institute of Pathology at Heidelberg University according to the respective criteria of the WHO classification during 06/2023 to 03/2025. Sample and data processing protocols were in accordance with the ethics committee of Heidelberg University (S-315/2020) and all methods were performed in accordance with the relevant guidelines and regulations. All relevant data are present within the paper and its supplementary information.

### Cohorts

**Fig. 2:**
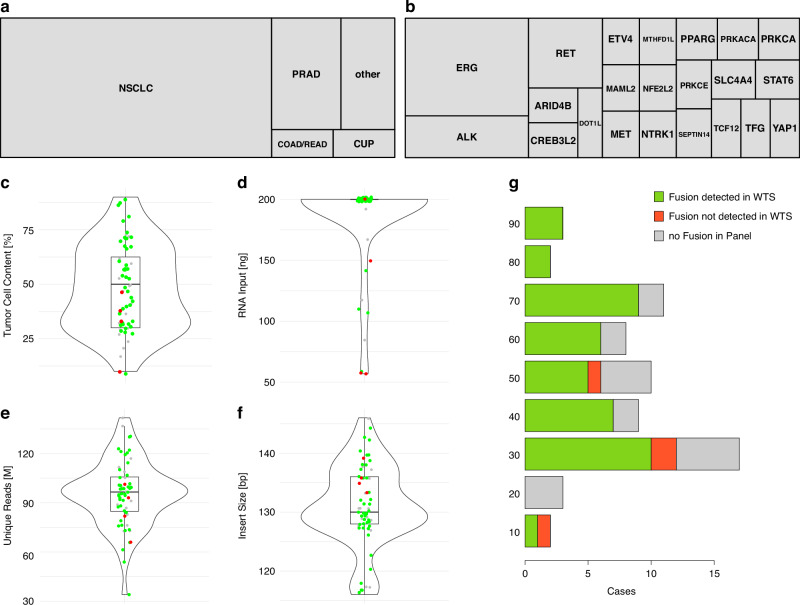
Comparison of fusion detection using whole transcriptome sequencing (WTS) and targeted RNA panels in the EC (*n* = 64). Tree maps representation the composition of cancer types (**a**) and oncogenic fusion partner (**b**) in the EC. **c**–**f** The violin and boxplots showing the distribution of different sequencing QC metrics, 48 of them positive for fusions. Sample distributions for QC metrics are shown in (**c**) by TCC; (**d**) RNA input amount [ng]; **e** Unique molecules sequenced; **f** Library insert sizes [bp]. Green dots indicate cases where a fusion was detected, red dots indicate cases where the fusion was expected but not detected, and grey dots represent cases with no fusion in the panel. **g** Bar chart summarizing the fusion detection results according to the TCC. Green bars represent cases where the fusion was detected using WTS, red bars indicate cases where the fusion was expected but not detected in WTS, and grey bars denote samples with no fusion detected in the panel. The four missed gene fusions, EML4::ALK, EGFR::EGFR, BRCA2::SLC4A4, and METΔex14, are highlighted in red. Color representation for plots C-G for correctly identified fusions are shown in green, correctly negative ones are shown in grey and red represents missed fusion calls.

The evaluation cohort (EC) consisted of 64 selected solid tumor samples previously sequenced with TSO500 RNA (*n* = 18) or Archer FusionPlex (Pan Solid Tumor v2 (*n* = 39), Archer Solid Tumor (*n* = 2), Archer Lung v2 (*n* = 5)), of which 48 samples were fusion-positive and 16 fusion-negative samples. The composition of tumor types is shown in Fig. 2a.

For the validation cohort (VC) (*n* = 357), samples that were analyzed using TSO500 RNA (*n* = 179) or Archer FusionPlex Archer Lung v2 (*n* = 2), Archer Lung (*n* = 2), Pan Solid Tumor v2 (*n* = 174) in the routine diagnostic workflow and had sufficient RNA amount for further testing were additionally analyzed by WTS.

The diagnostic WTS only cohort consisted of all diagnostic solid tumor tissue samples (*n* = 812) from July 2024 until March 2025 meeting the thresholds of at least 40% TCC, 50 ng total RNA input, 50 M unique reads and median insert size of at least 100 bp. In this timespan, a total of 1235 clinical RNA samples were analyzed, of which 423 were subjected to a targeted panel.

### RNA isolation

Tumor areas were marked on HE-stained slides and corresponding tissue areas were macrodissected, marking the tumor area and scratching material from subsequent unstained 5-10 μm thick FFPE-sections. Tumor cell content (TCC) was estimated by an experienced pathologist using a microscope.

RNA extraction was conducted automatically using the Maxwell RSC FFPE RNA Kit on a Maxwell RSC Benchtop Instrument (Promega, Madison, WI, USA), in accordance with the manufacturer’s instructions. The RNA concentration was measured fluorometrically (QuBit 2.0 RNA high sensitivity kit, Thermo Fisher Scientific, Waltham, MA, USA) following the manufacturer’s instructions. RNA integrity was assessed using the RNA ScreenTape assay on an Agilent 4200 TapeStation System (both Agilent, Santa Clara, CA, USA).

### Library preparation and sequencing

For library preparation 50–200 ng RNA were used as input. Whole transcriptome libraries were generated with Illumina TruSeq Stranded Total RNA Prep with Ribo-Zero Plus Kit with integrated rRNA depletion (Illumina, Madison, WI, USA), according to the manufacturer’s instructions.

Complementary RNA-based fusion analyses were conducted with either the hybrid-capture method using the TSO500 RNA Panel (Illumina) or an anchored multiplex PCR approach by Archer FusionPlex Pan SolidTumor v2 Panel (IDT, Boulder, CO, USA) in keeping with the manufacturer’s instructions. Subsequently, WTS and TSO500 RNA libraries were sequenced on a NovaSeq 6000 and the Archer FusionPlex libraries on a NextSeq550Dx (both Illumina) according to the manufacturer’s instructions.

### Bioinformatic pipelines

#### Panel-based fusion detection

Archer Analysis v6.2.3 was employed for fusion detection using its proprietary pipeline. The minimum read count threshold was set to 10; all fusions and splicing events were reviewed and validated by an expert.

The TruSight Oncology 500 v2.2.1 Local App RNA pipeline was executed. Fusion calls were filtered using a read count threshold of 10. All fusions and splicing events were reviewed and validated by biologists with multiple years of experience in molecular diagnostic analysis.

### WTS-pipeline

The DRAGEN (Dynamic Read Analysis for GENomics) pipeline (version 4.1.7) (Illumina) was used for alignment, gene quantification, and fusion detection. RNA-seq reads were aligned to the reference genome (hg19) using the DRAGEN RNA module with default parameters. Gene fusion detection was performed using DRAGEN’s built-in fusion caller, applying a minimum fusion read support threshold of 1 read and a gene-set filter (Supplementary Table 1), all fusions were  reviewed and validated by an expert.

#### Gene fusion detection

ARRIBA [21] v2.4.0 was utilized for fusion detection and visualization. BAM files from DRAGEN pipeline output were then analyzed using ARRIBA with default parameters, applying a minimum fusion read support threshold of 1 read and gene-set filter (Supplementary Table 1). All fusions were reviewed and validated by an expert.

#### Alternative splicing events

CTAT-Splicing (version v0.0.3) was used for splicing event detection [22]. The pipeline utilized DRAGEN BAM files as input and performed splicing analysis against a curated reference dataset. Differential splicing events were detected using filtering criteria: minimum read support of 1 and gene-set filter (Supplementary Table 1), all splicing events were reviewed and validated by an expert.

#### Imbalance assay

Unbalanced expression within a gene (5’ vs 3’ region) can indicate the presence of a gene fusion in the absence of split reads or discordant mates [23]. Using the imbalance assay, the number of stranded reads and the number of splice junctions mapping to the gene were counted for the 5’ part of the gene and compared to the 3’ part, for recurrent breakpoints [24] (Supplementary Table 2) or for automatically defined breakpoints based on expression. Automatic breakpoint determination was implemented by fitting a linear model with the stepmented function from the segmented package in R [25, 26].

#### Detection and association of non-human sequences

The Kraken 2 pipeline was used to screen all RNA sequences for taxonomic classification using exact k-mer matches with default options and the k2_pluspf_16gb_20240112 database(27, https://benlangmead.github.io/aws-indexes/k2).

#### Pathway analyses

Pathway associations with gene fusions were evaluated using the FusionPathway methodology [27], which infers functional consequences of fusion events based on alterations in protein–protein and protein–DNA interaction networks. The protein domain composition (domains retained or lost in a fusion) was identified from fusion annotations provided by ARRIBA in the WTS-only cohort. Network-based prioritization of functionally relevant pathways was conducted using the Random Walk with Restart (RWR) algorithm, implemented via the FusionPathway R package (v1.0.0) using default parameters [27]. For pathway enrichment, curated gene sets were obtained from the Molecular Signatures Database (MsigDB) [28], specifically including collections from Gene Ontology Biological Processes (C5: BP), KEGG MEDICUS (C2: KEGG), Hallmark gene sets (H), and Oncogenic Signatures (C6). These gene sets were used as input for enrichment analysis with the fgsea package (v1.32.4) [29]. Gene-level count data were normalized using variance stabilizing transformation (VST) from the DESeq2 package (v1.46.0) to explore gene expression profiles [30].

#### Statistical analyses and visualization

RNA feature assignments were done with featureCounts [31] and R packages Treemaps were created with the treemap package [32].

Fastq files of WTS were analyzed with Kraken v2.1.2 [33] using the “k2_pluspf_16gb_20240112” database. For visualization of metagenomics, output of Kraken was used with KronaTools v2.8.1 [34]. For visualisation and revision of RNA sequences, IGV v2.9.1 was used [35], plots were generated in R with the packages GGPLOT [36] and complex heatmap [37].

## Results

### Assessing QC parameters for fusion and splice variant calling using WTS in a diagnostic set-up

For the implementation of WTS to determine fusion and splice variants in routine diagnostics, an evaluation cohort (EC) of 64 clinical FFPE samples was selected in a first step. All 64 samples (Fig. 2a-g) were previously sequenced with a targeted panel-based assay (hybrid–capture based TSO500 RNA; AMP-based Archer Fusion Plex Pan Solid Tumor v2) for fusion calling established in our lab. In contrast to those assays the now applied WTS approach is based on a stranded protocol with rRNA depletion. For comparison of the different methods, the analysis of the fusion and splice variant calling using WTS was restricted to genes included in the two targeted panels (Supplementary Table 1). Fusion and splice variant calling in the WTS samples was initially performed using the three bioinformatic pipelines Arriba, Dragen and the CTAT-splicing in parallel (Fig. 1).

Of the samples with known fusions (*n* = 48) in the EC, 44 (92%) were successfully identified with the WTS approach. All fusion negative samples (*n* = 16) were negative for the genes tested with WTS (Fig. 2c-g).

The selected samples of the EC consisted predominantly of non-small cell lung cancer (NSCLC, *n* = 44), prostate adenocarcinoma (PRAD, *n* = 10), colon/rectum adenocarcinoma (COAD/READ, *n* = 2) and cancer of unknown primary (CUP, *n* = 2) (Fig. 2a). The largest proportion of detected fusions involved *ERG* followed by *RET* and *ALK* (Fig. 2b).

Four fusion-positive samples were not identified by our initial WTS analysis pipelines: an EGFR::EGFR exon duplication (case N1), a BRCA2::SLC4A4 fusion (case N2), a MET Δex14 (case N3) and an EML4::ALK (case N4) fusion (Fig. 2g).

A more detailed analysis of the QC parameters of the four false-negative samples revealed a TCC of 50%, 30% (two samples), and 10% respectively (Fig. 2c). Regarding the RNA input amount, two samples were at the lower end with 50 ng, one had 150 ng and one the maximum input amount of 200 ng (Fig. 2d). The distribution of unique mapped reads for all samples spanned from 32 M to 140 M reads with a median of 96 M reads. The four failed samples ranged from 70 M to 100 M reads close to the median (Fig. 2e. For the mean insert size of the fragments, all four failed samples were above the median of 129 bp ranging from 133 bp to 138 bp with an overall distribution from 116 bp to 152 bp (Fig. 2f). The amount of RNA input above 50 ng, the number of unique reads and insert length did not generally affect the successful fusion detection using the WTS approach in this cohort.

### A bioinformatic imbalance assay supports fusion calling

Next, we investigated the failed detection of N1-N4. Case N1 is a duplication of kinase domain exon 18-25 in *EGFR* with a TCC of 30%. Therefore, rather than being a fusion or splicing alteration, it is the result of a structural variant. At the DNA level, this is most accurately classified as a copy number variation (CNV) event. At the RNA level, this leads to reads spanning from exon 25 to exon 18, as can be seen in the WTS data (Supplementary Fig. 1). However, the alteration was not identified because it falls outside the scope of the bioinformatic tools used. N2 is a deleterious *BRCA2* translocation nonsense-mediated mRNA decay [38] with a TCC of 30%, the MET Δex14 in N3 was not detected at a TCC of 10%.

**Fig. 3:**
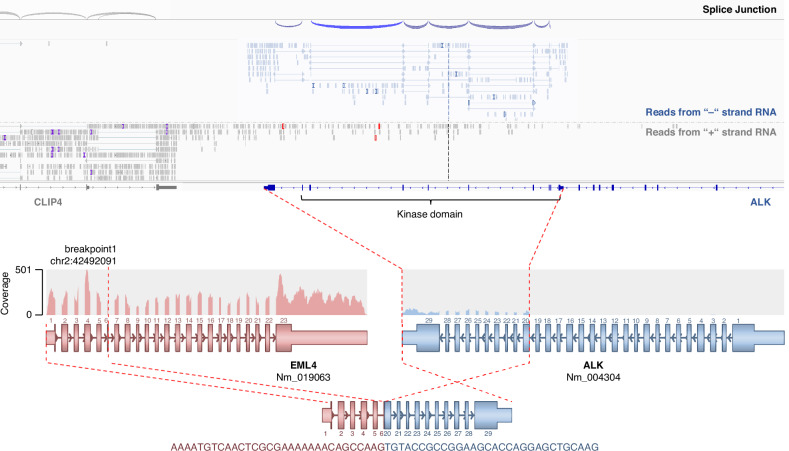
A—Imbalance Assay for EML4::ALK fusion. Top: IGV representation of the missed EML4::ALK fusion in WTS: +/- represent the positive/negative strand based on stranded protocol information. Spliced reads for ALK on the + strand are depicted as grey lines connecting the grey cigars, spliced reads of the—strand are depicted in blue. The splice junction track on top represents splice junctions on the + strand in blue and on the—strand in red. Unspliced reads originating from *CLIP4* reaching into the kinase domain of ALK on the—strand. Bottom: Visualization of the EML4::ALK fusion with the coverage track on top, the exon and breakpoint representation below and the resulting fusion transcript on bottom.

The EML4::ALK fusion in case N4 had a TCC of 50%. No reads specific for an ALK fusion could be detected. Reads mapping to the exons coding for the ALK protein kinase domain were present, but not for the upstream exons (Fig. 3). As shown before, unbalanced transcript expression is a predominant feature of fusion transcripts [23]. In the case of ALK, which is not expressed in adult wild-type tissue [39], expression starting after exon 19, where most of ALK translocations occur [40], could be indicative of an ALK fusion. Therefore, an imbalance assay was developed as an additional approach to infer the presence of a gene for the indication of fusions. In the case of the detection of an unbalanced expression, additional confirmation by an orthogonal test can then be performed in a clinical diagnostic workup.

Therefore the mean coverages between the exons on both sides of the most common breakpoints from Mitelman Database [24] and in-house database in the gene-set (Supplementary Table 2) as well as the spliced reads on the gene-specific strand were counted and visualized in IGV (Fig. 3). For this specific case, considering the gene specific strand for the *ALK* transcript, exons 1–19 showed a mean coverage of 0.03x and exon 20–34 of 7.37x. This represents a fold-change of 59.2. Second, the counts for these regions showed 84 vs. 0 spliced reads on the gene specific strand. Both aspects suggest a strongly increased transcriptional activity for the 3’ part of ALK. Of note, by investigating the WTS data of this *ALK* fusion, plenty of unspliced RNA on the opposite strand to the *ALK* transcript could be seen originating from *CLIP4* pre-mRNA. Hence, the strand identity has to be considered for the mean coverage, to prevent false positive counts based of the antisense strand. The count of splice junctions on the other hand was not biased by antisense reads.

In summary, taking the results of our EC into account we defined the following thresholds of pre-sequencing QC metrics for calling of fusions and alternate splicing events in WTS: TCC of 40% or more and a total input amount of at least 50 ng of RNA. For post-sequencing QC metrics, we established a cutoff for valid samples of at least 50 M unique reads and median insert sizes of at least 100 bp. Significantly different transcriptional activities in genes with known therapeutic targets for a pre-defined gene-set (Supplementary table 2), like *RET*, *ROS1*, and *ALK* missing unambiguous split reads, were subjected for reanalysis with a targeted panel.

### Validation of the WTS fusion and splice variant calling pipeline for diagnostic use

**Fig. 4:**
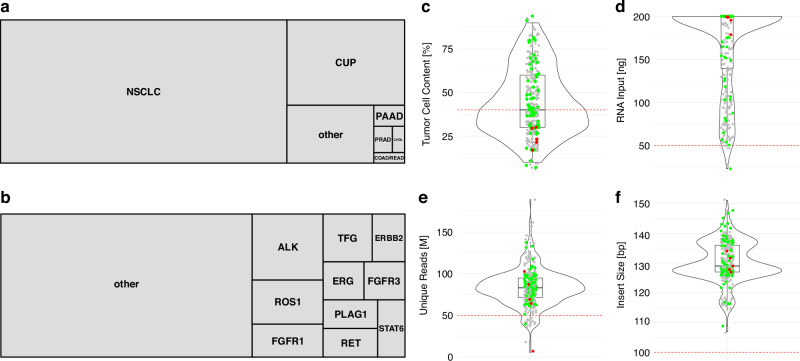
Fusion detection using WTS in the validation cohort (*n* = 357). Tree maps representing the composition of cancer types (**a**) and oncogenic fusion partner (**b**) in the VC. **c**–**f** Violin plots with embedded boxplots showing the distribution of sequencing QC metrics for all samples. Sample distribution for QC metrics are shown in (**c**) by TCC; **d** RNA input amount (ng); **e** Unique molecules sequenced; **f** Library insert sizes (bp). Green dots indicate cases where a fusion was detected, red dots indicate cases where the fusion was expected but not found, and grey dots represent cases with no fusion in the panel. The red dashed lines mark thresholds for relevant sequencing metrics of 40% TCC, 50 ng RNA input, 50 M unique reads and 100 bp insert size.

Following the analysis of the EC results and implementation of the imbalance assay, the approach was validated by parallel analysis of a set of routine diagnostic samples (*n* = 357; validation cohort (VC); Fig. 4a-f) with a targeted fusion panel and WTS. The predominant cancer types were NSCLC (*n* = 253; 70.9%) followed by CUP (*n* = 62; 17.4%) and other solid tumor types, grouped in other (*n* = 31; 8.7%).

Of the 357 VC samples, 131 (37%) had a TCC below 40% (36.7%), 4 samples were sequenced with a total RNA input below 50 ng (1.1%), 21 samples did not reach the 50 M unique reads after collapsing (5.9%) (Fig. 4c-f). Thus, 144 samples did not meet the pre-defined QC criteria (40.3%), while 213 samples (59.7%) met all QC criteria, applying the TCC threshold of 40%, over 50 ng total RNA input, more than 50 M unique sequenced reads and a median insert size of above 100 bp.

Applying the QC-metrics we established resulted in a 100% match in fusion calls between the WTS approach and the targeted fusion panels. Of note, even if all thresholds were disregarded, 352 of the 357 samples (98.6%) were still identified correctly. Sixty-nine out of 74 fusions were called correctly (93.2%) when compared with the results obtained from the targeted fusion panel. All of the five samples with false negative results had a TCC of 30% or 20%, falling below the defined threshold of 40% (Fig. 4c). The further QC metrics of RNA input amount, unique reads and insert sizes (Fig. 4c-f) did not reveal any further need for adjustments of these parameters and were in agreement with the QC data from the EC (Fig. 2c-f).

WTS did not identify additional fusions beyond those already identified by targeted panel sequencing for the validated gene-set (Supplementary Table 1).

### WTS in clinical practice

Following the successful validation process, the application of WTS for diagnostic gene fusion detection was initiated. During the period spanning from July 2024 until March 2025, a total of 812 clinical samples, which met our QC parameter, were subjected to sequencing and analysis for fusion detection through the utilization of WTS. Specimen type was available for 339 samples, consisting of 193 biopsies (56.9%) and 146 resections (43.1%).

**Fig. 5:**
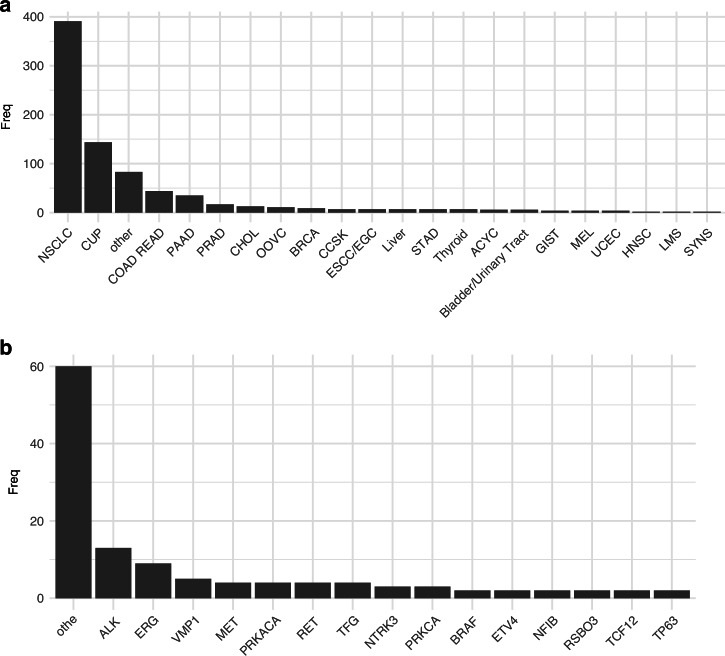
Overview of the clinical WTS cohort after adaptation in diagnostics. **a** Bar chart representing the cancer type frequencies analyzed (*n* = 812). **b** Bar chart representation of validated fusions (*n* = 121).

The 812 clinical WTS samples included a diverse range of tumor types (Fig. 5a). NSCLC was the most prevalent cancer type with 391 samples (48.2%), followed by CUP (*n* = 144; 17.7%). We detected 121 fusions with a diverse range of fusion with ALK and ERG being the two most frequently detected fusion partners (Fig. 5b). Turnaround times for WTS and targeted approaches were comparable.

In the same time span, 423 of the 1235 diagnostic samples (34.3%¸78% NSCLC (*n* = 330) had to be analyzed with one of the targeted panels as they did not meet our QC parameters.

### Leveraging WTS data beyond fusions and splice variant calling

The use of WTS in diagnostics can provide additional potential valuable information for the patient.

To further improve molecular profiling, an expanded gene list (Supplementary Table 3), comprising regulatory genes, oncogenes, and tumor suppressor genes, was employed to detect fusion transcripts beyond the scope of targeted approaches. A stricter threshold of 10 reads was applied to identify the most frequently altered transcripts for the whole WTS cohort.

**Fig. 6:**
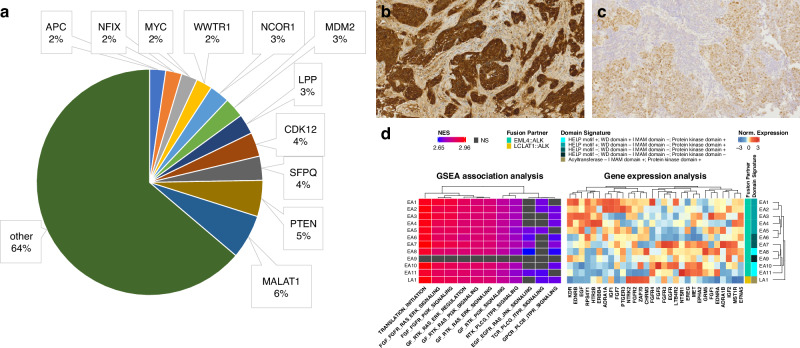
Further applications of WTS in the diagnostic setup. The Pie Chart in **a** shows the most frequent additionally found translocations (*n* = 174) in all WTS samples (*n* = 1233) not covered in the targeted panels with at least 10 reads. Genes found less than 4 times are grouped in ”other”. Two exemplary samples of metagenomic analysis are shown in (**b**) for HPV including Immunohistochemical stain of p16-positive squamous cell carcinoma(tonsil) and (**c**) for EBV including EBV in-situ hybridization of EBV-associated nasopharyngeal carcinoma (nonkeratinizing squamous cell carcinoma). **d** Composite heat map integrating Gene Set Enrichment Analysis (GSEA) and gene expression profiles of 12 ALK fusion-positive samples. Samples are annotated by fusion partner (EML4::ALK or LCLAT1::ALK) and associated domain signatures (retained [+] or lost [−] protein domains). The left panel (GSEA Association Analysis) shows normalized enrichment scores (NES) for pathways significantly enriched (padj ≤ 0.05) across samples. For each sample, the top 10 pathways by NES were selected, and their union was used for visualization. Pathway enrichment is color-coded from purple (suppressed activity) to red (increased activity), with black denoting non-significant results (NS). The right panel (“Gene Expression Analysis”) displays variance-stabilized, z-score normalized expression of the 2000 most variable genes across the samples. Matching row annotations highlight the relationship between expression profiles, pathway activation, and fusion-specific domain alterations.

Among these, MALAT1 was the most prevalent (*n* = 11), followed by PTEN (*n* = 9), and SFPQ and CDK12 (both *n* = 6) (Fig. 6a). These findings reveal further alterations that could be clinically relevant, such as the loss of the tumor suppressor PTEN due to truncation of the transcript after exon 2 in a case of a pulmonary adenocarcinoma (Supplementary Fig. 2). This variant resembles the effect of a deleterious PTEN mutation, causing a loss of function. This loss of the negative regulator of the PI3K/AKT signaling pathway can act oncogenic and argues for the discussion of a potential treatment with AKT inhibitors, such as Capivasertib. Which is approved, in combination with Fulvestrant, for the treatment of adult patients with oestrogen receptor (ER)-positive, HER2-negative, locally advanced or metastatic breast cancer with one or more PIK3CA/AKT1/PTEN alterations, following recurrence or progression of the disease during or after endocrine therapy.

As a second representative case, an adenoid cystic carcinoma with a MYB::NFIB gene fusion is presented (Supplementary Fig. 1). While MYB::NFIB fusions are a key molecular hallmark of adenoid cystic carcinoma, the structure of this specific fusion is remarkable, as a 1.3 kb intergenic insert was detected, connecting the two partners in this bridged fusion. It is evident that the canonical splicing donor site referring to exon 9 (NM_005375.4) is not utilised in the fusion transcript. However, transcription persists for a region of over 450 bp within intron 9, extending over the potential breakpoint (Chr9:135518908), into an intergenic region (Chr9:15337270-15338597), where de novo splice sites are employed. A similar event can be observed for the second potential breakpoint, leading back to NFIB (Chr9:14088338) shortly before the canonical splice acceptor site of exon 11 (NM_001190737.2), where no splicing can be seen (Supplementary Fig. 3). The intersection and the altered splicing events can complicate the detection of the fusion with a targeted approach based on amplicon, single primer detection, or hybrid capture enrichment strategies, making it difficult or even impossible to detect. WTS using rRNA depletion allows the investigation of the transcriptome for the presence of non-human transcripts, for example, from pathogens. Figure 6b, c display two representative results. In the first case a squamous carcinoma of the tonsil, which was p16-positive by immunohistochemistry, 2485 reads for human papillomavirus 16 could be identified (Fig. 6b). In another case 35190 reads from *human gamma herpesvirus 4* (EBV) were detected, in line with the pathological report of an EBV associated non-keratinizing nasopharyngeal carcinoma, positive for Epstein-Barr encoding region detected by in situ hybridization (Fig. 6c).

Furthermore, the human transcripts of the WTS data inherently encompass additional information beyond the mere gene fusion status, which can be utilized for further analysis. To explore the functional impact of ALK fusion events at both the signaling and transcriptional levels, we conducted an integrative analysis combining protein domain composition signatures with gene expression data from 12 ALK fusion-positive samples (Fig. 6d). Hierarchical clustering of gene expression patterns (right panel) revealed distinct gene expression profiles. Furthermore, we identified frequently enriched pathways (padj ≤ 0.05) associated with receptor tyrosine kinase (RTK) signaling and the downstream RAS-MAPK and PI3K cascades, highlighting their central role in ALK-driven oncogenic processes (Fig. 6d).

## Discussion

Cancer diagnostics requires continuous innovation to improve diagnostic yield and inform treatment decisions optimally. For oncogenic gene fusions, a comprehensive analysis of novel and recurrent gene fusions is needed, thereby enhancing the efficiency of the diagnostic process. Further, clinical diagnostic workflows must be robust and reproducible. Therefore, when implementing novel techniques, implementation must be carefully planned and supervised. In this study, we describe the implementation of WTS for the detection of somatic gene fusions and splice variants from FFPE samples of solid tumors. As the method of choice rRNA depletion was selected due to its ability to provide a more comprehensive view of the transcriptome. Unlike Poly(A) selection, it does not rely on polyadenylation, making it suitable for analyzing degraded FFPE-derived RNA. Additionally, it allows for the detection of both coding and non-coding RNAs, as well as potential non-human RNA, unlike WTS by exome enrichment [41–43]. The primary limitation of the depletion-based approach is its requirement for increased sequencing depth to get comparable exonic coverage relative to the hybrid capture method. For example, when determining the distribution of reads separated by coding and non-coding features for the EC, a mean of 49% of the reads could be attributed to protein coding sequences (Supplementary Fig. 4 and Supplementary Table 4).

The evaluation of WTS for fusion and splice variant detection in clinical FFPE samples demonstrated a high consensus of known fusions, with 44 out of 48 fusions (92%) successfully identified in the initial evaluation cohort. However, in four samples, no split or discordant mate reads specific for the respective fusion were found and further analyses revealed that three of these samples had TCC of 30% and below, while the fourth had a TCC of 50% with an EML4::ALK fusion. Other QC metrics, such as RNA input amount, unique reads, and insert sizes, had no or only minor influence on the performance. Of note, sample quality was measured by insert size rather than RIN, as we considered this post-sequencing QC parameter to be more informative for assessing RNA quality in FFPE-derived samples. None of the samples in the EC and VC had mean insert size below 100 bp (Figs. 2f and 4f). Interestingly, some gene fusions could be detected down to 10% TCC, indicating potentially different TCC limits for different fusions due to their overall expression. However, for clinical samples, strict thresholds were chosen for general fusion detection and to account for possible overestimations in TCC estimate [44].

To address the known limitations in fusion calling [21], an imbalance assay was introduced as an additional safeguard and to improve sensitivity for fusion detection [23]. This assay analyzes transcriptional activity by comparing the stranded exon coverages on both sides of a recurrent breakpoint, as well as the number of spliced reads, as demonstrated with the *ALK* fusion case (Fig. 3). This approach enhances fusion detection sensitivity, particularly for cases where no unambiguous split reads for the fusion partner are detected. The imbalance assay is most important for genes with low or no transcriptional activity, showing an imbalance and thus provides an additional layer of detection for therapeutically relevant fusions. In such cases an orthogonal method like targeted panel, FISH or CISH needs to be used to confirm the fusion/translocation and/or to reveal the fusion partner [4, 45].

Following the implementation of the imbalance assay, a larger cohort of 357 clinical RNA samples was analyzed for validation. The application of the 40% TCC threshold ensured reliable fusion detection for 226 of the 357 cases (63%). It is evident that the relatively high TCC cutoff is largely attributable to the rRNA depletion strategy, which trades sensitivity for broader data acquisition. In the validation cohort and during the implementation of clinical WTS, 37% and 34% of cases, respectively, exhibited a tumor fraction below 40%. Consequently, these cases necessitated a transition to targeted testing. Although WTS-based fusion detection was occasionally feasible in samples with lower tumor content, a conservative cutoff was applied to minimise false negatives. Increasing the sequencing depth or utilising exome enrichment-based WTS has the potential to enhance sensitivity. However, it should be noted that these approaches would result in a substantial increase in costs. Given the potential for a low tumor content to influence downstream analyses, such as gene expression, targeted fusion testing was utilised as a backup approach for these samples.

In this cohort, five fusions failed to be detected due to low TCC. No further limitations concerning RNA input amount, unique reads, or insert size arose, confirming the robustness of the predefined QC metrics. Importantly, 98.6% of the samples were correctly identified even when all thresholds were disregarded, highlighting the overall effectiveness of WTS in fusion detection.

Our study demonstrates that TCC and sufficient unique sequences, in this study 50 M unique reads for the validated genes (Supplementary Table 1), are the most critical parameter affecting gene fusion detection. The introduction of a minimum TCC threshold of 40% for reliable detection was supported by additional clinical samples, reinforcing the importance of setting rigorous QC criteria. In two clinical practice cases, no split reads and one split read was found for an ALK translocation, but could be identified by the introduction of the imbalance assay.

FFPE samples present inherent challenges due to RNA degradation and chemical cross-linking during the fixation process. Targeted sequencing panels such as Archer FusionPlex and TSO500 RNA are optimized for these conditions, but they are also limited to detecting fusions based on the regions of interest included in the respective panel design. This can be attributed to the overall sequencing coverage of a targeted approach, which facilitates deeper sequencing for the specific target regions at a reduced cost. Although WTS has lower sensitivity this approach is unbiased and genome-wide. This allows for the detection of novel or unexpected fusion events, including those involving non-coding regions or complex rearrangements. Which is particularly valuable in cancers with complex fusion landscapes, such as lung cancer, biliary tract cancers as well as CUPs, where novel fusions can serve as key biomarkers for targeted therapies. Furthermore, this approach enables the detection of tumor suppressor loss-of-function transcripts, which might be missed by DNA analysis limited to coding regions.

Considering the variety of WTS approaches, a stranded RNA depletion assay was selected in preference to an exome enrichment assay, not for the advantage of the identification of fusion genes, but rather for the purposes of further analysis. In fact, enrichment based WTS will show fewer intronic reads due to premature RNA than an rRNA depletion assay. The resulting higher overall on-target rate would be beneficial for the detection of fusion reads. However, when considering gene expression profiling, rRNA depletion has the advantage that read counts are less biased due to differences in hybridization efficiency. It also has the potential to identify pathogens, such as oncogenic viruses, to complement diagnosis. PolyA enrichment is not applicable for fusion detection based on the challenging and highly degraded FFPE derived RNA and short read sequencing.

A stranded protocol is superior to an unstranded approach for unambiguous signals from the reading strand, which is essential for the imbalance assay and gene expression profiling in general.

Our WTS approach provides simultaneous insights into global and quantitative gene expression, alternative splicing, and pathway analysis. This offers a more comprehensive molecular profile of the tumor, which already is used for some cancer types, especially tumors with complex splicing patterns or transcriptional dysregulation, such as hematological malignancies, but also prostate cancer, gliomas, melanoma, triple-negative breast cancer and NSCLC [11, 18, 19]. The integrative analysis demonstrated that both fusion partner identity and domain composition shape pathway activity and global gene expression in ALK fusion-positive tumors. The observed diversity in transcriptional and signaling profiles highlights substantial molecular heterogeneity driven by distinct fusion events. Thus, this integrative bioinformatic analysis strategy may enhance the functional interpretation of gene fusions and support more tailored clinical decision-making.

Overall, this study underscores the strengths and limitations of WTS for fusion detection in clinical FFPE samples, including biopsies. The application of a strict TCC threshold and complementary imbalance assays improves the reliability of WTS, making it a valuable tool for personalized cancer diagnostics. Future research should focus on optimizing RNA extraction and library preparation techniques to further enhance WTS performance in FFPE-derived RNA samples. Additionally, continued refinements in bioinformatic pipelines will be necessary to improve fusion detection sensitivity, particularly in low-TCC samples.

In conclusion, although targeted panels remain effective for identifying known fusions, whole transcriptome sequencing offers a broader and more versatile approach, capable of detecting novel fusion events while simultaneously delivering comprehensive transcriptomic insights, including pathway activation and identification of pathogens. The implementation of stringent QC thresholds, in combination with advanced analytical approaches such as the imbalance assay, enhances the applicability of WTS in clinical practice. By refining workflows and incorporating additional validation measures, WTS has the potential to improve molecular diagnostics and guide precision oncology treatments effectively.

## Supplementary information

Supplemental legends

Supplemental Figure 1

Supplemental Figure 2

Supplemental Figure 3

Supplemental Figure 4

Supplemental Table 1

Supplemental Table 2

Supplemental Table 3

Supplemental Table 4
