# Correction: Diagnostic whole transcriptome sequencing in a series of 1233 FFPE solid tumor samples

**DOI:** 10.1038/s41416-026-03360-x

**Published:** 2026-03-02

**Authors:** Markus Ball, Susanne Beck, Darius Wlochowitz, Tina Fuchs, Katja Lorenz, Christiane Zgorzelski, Alejandro Pallares Robles, Michael Allgäuer, Anna-Lena Volckmar, Hannah Goldschmid, Iordanis Ourailidis, Regine Brandt, Petros Christopoulos, Michael Thomas, Huriye Seker-Cin, Annette Fink, Fabian Schnecko, Olaf Neumann, Michael Menzel, Martina Kirchner, Thoas Fioretos, Peter Schirmacher, Solange Peters, Jan Budczies, Albrecht Stenzinger, Daniel Kazdal

**Affiliations:** 1 Institute of Pathology, Heidelberg University Hospital, Heidelberg, Germany; 2 Translational Lung Research Center (TLRC) Heidelberg, German Center for Lung Research (DZL), Heidelberg, Germany; 3 Department of Medical Oncology, Thorax Clinic, Heidelberg, Germany; 4 Division of Clinical Genetics, Department of Laboratory Medicine, Lund University, Lund, Sweden; 5 Department of Clinical Genetics, Pathology and Molecular Diagnostics, Office for Medical Services, Region Skåne, Lund, Sweden; 6 Oncology Department, CHUV, Lausanne University, Lausanne, Switzerland; 7 German Cancer Consortium (DKTK), Heidelberg, Germany; 8 Center for Personalized Medicine Heidelberg (ZPM), Heidelberg, Germany

**Keywords:** Predictive markers, Medical genetics, Molecular medicine, Cancer

Correction to: *British Journal of Cancer* 10.1038/s41416-025-03307-8, published online 14 January 2026

In this article, the author’s name, Olaf Neumann, was incorrectly written as Olaf Neuman.

The original article has been corrected.

