# Supplementary material for: Diagnostic whole transcriptome sequencing in a series of 1233 FFPE solid tumor samples
Source: Br J Cancer. 2026 Jan 14;134(7):1101–10. doi: 10.1038/s41416-025-03307-8 (PMC12996614; doi:10.1038/s41416-025-03307-8)
Supplement: Supplementary file 1 — Supplemental legends [file 41416_2025_3307_MOESM1_ESM.docx]

Supplementary Table 1:

Genes included in validation for WTS in diagnostics.

Supplementary Table 2:

Known fusion breakpoints

Supplementary Table 3:

Extended gene list

Supplementary Table 4

Summary for sequencing reads mapped across 64 samples of EC, for categorized total mapped reads into genomic features, including protein coding, unassigned multi-mapping, non-coding, other, and lincRNA. For each category, the mean, median, standard deviation (SD), minimum (Min), and maximum (Max) read counts. The percentage of mapped column indicates the mean proportion of each category relative to the total mapped reads.

Supplementary Figure 1:

IGV visualization of the EGFR locus. The green arrow indicates a cluster of discordant read pairs (green horizontal lines) that span exon 18 to exon 25. These reads show a changed orientation and an increased insert size compared to the reference genome, providing evidence for a structural variant in this region.

Supplementary Figure 2:

Detection and Functional Impact of PTEN::RNLS Fusion. This figure displays the PTEN::RNLS fusion transcript as identified by the ARRIBA bioinformatics pipeline. The fusion event exhibits opposing transcriptional start sites, leading to a loss-of-function for the *PTEN* gene.

Supplementary Figure 3:

Bridged MYB::NFIB Gene Fusion. Top: The two Arriba plots detail the structure of a bridged MYB-intergenic-NFIB fusion. The MYB gene on chromosome 6 fuses to a 1.3 kb intergenic region on chromosome 9. This intergenic region subsequently fuses to the NFIB. Coverage is shown above the gene annotations. Bottom: Integrative Genomics Viewer (IGV) screenshots show sequencing reads supporting the fusion transcripts.

Supplementary Figure 4:

Violin plots display the distribution of log10-transformed read counts for various stages of read assignment across all samples. Major categories shown are Total Reads (green), Total Unassigned Reads (orange), and Total Assigned Exonic Reads (dark purple). The Subset of Unassigned Total includes reads categorized as unassigned_nofeatures, unassigned_multimapping, and unassigned_other, the Subset of Assigned Exonic Reads includes reads categorized as assigned_protein_coding, assigned_noncoding_total, and assigned_other3.
