# Supplementary figures and images for: Diagnostic whole transcriptome sequencing in a series of 1233 FFPE solid tumor samples
Source: Br J Cancer. 2026 Jan 14;134(7):1101–10. doi: 10.1038/s41416-025-03307-8 (PMC12996614; doi:10.1038/s41416-025-03307-8)

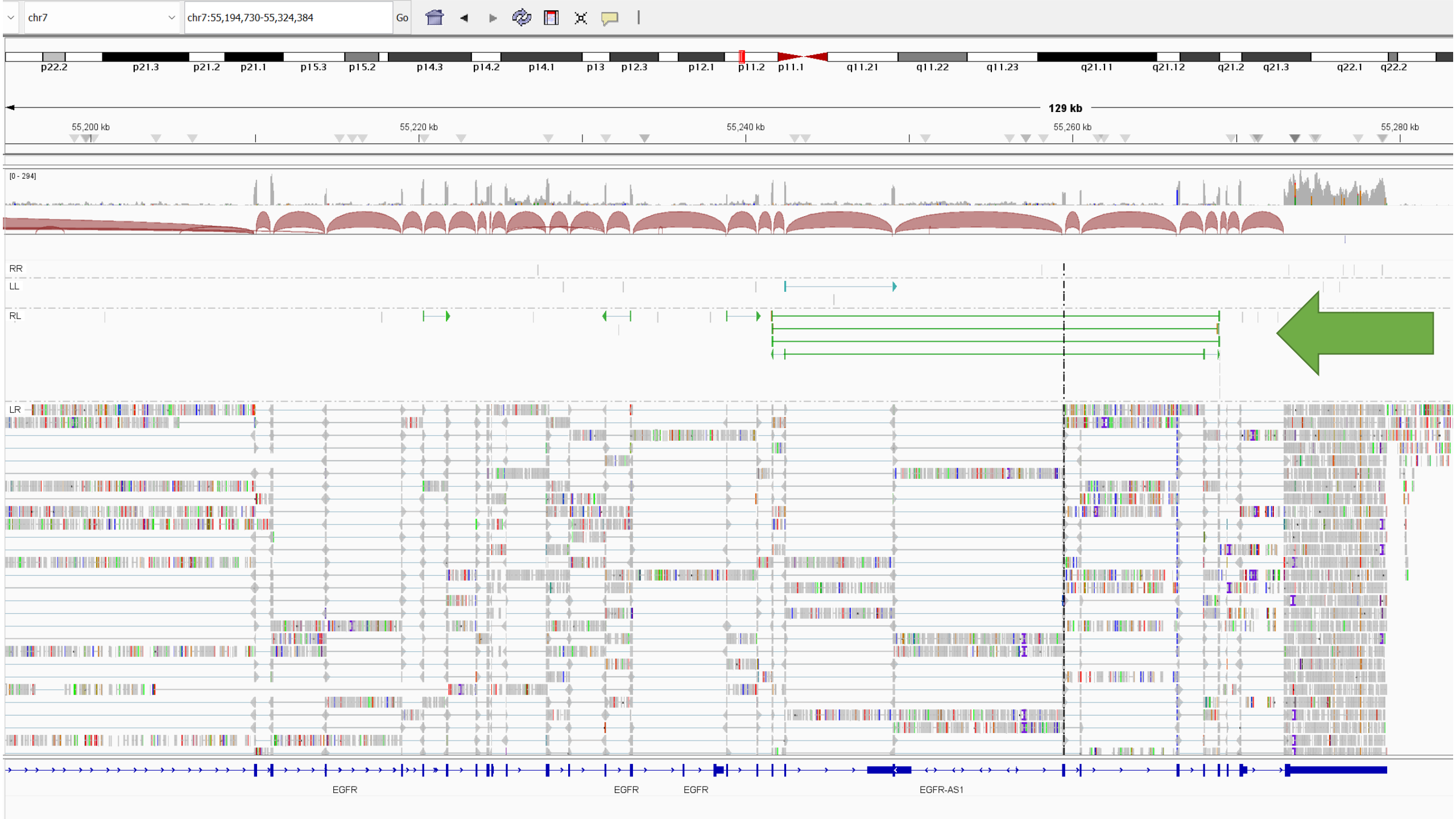

Supplement: Supplementary file 2 — Supplemental Figure 1 [file 41416_2025_3307_MOESM2_ESM.pdf]

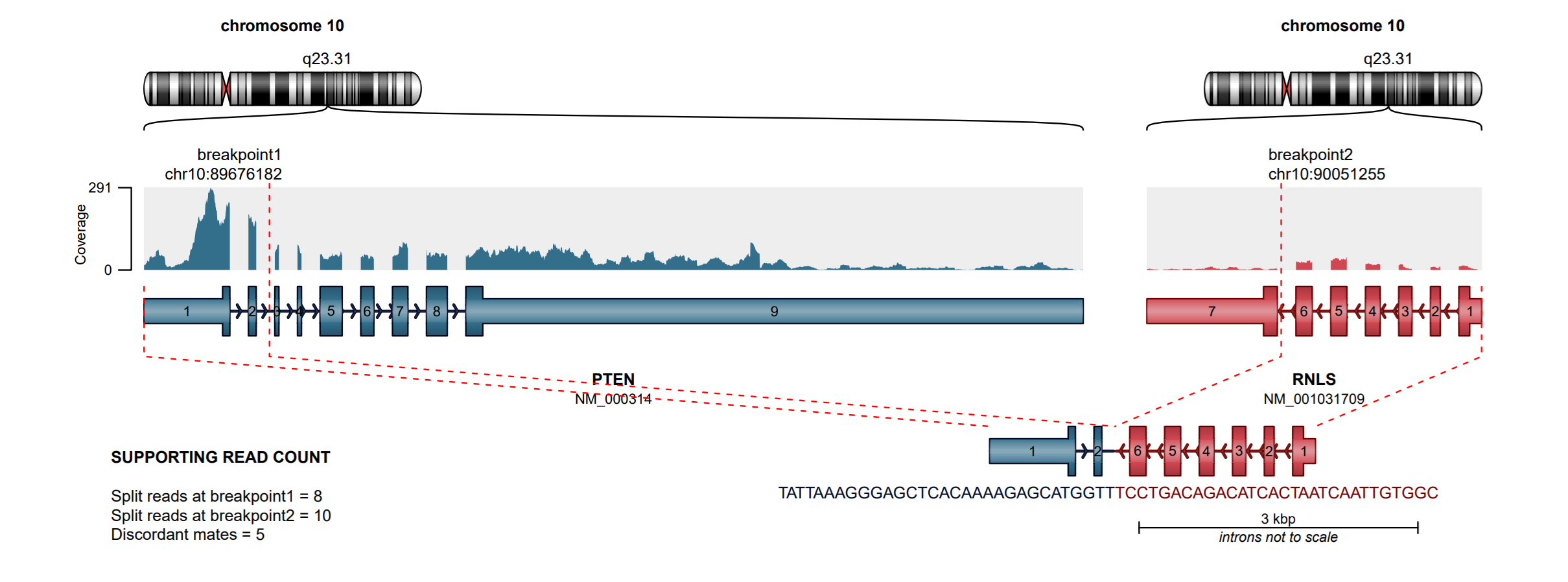

Supplement: Supplementary file 3 — Supplemental Figure 2 [file 41416_2025_3307_MOESM3_ESM.tif]

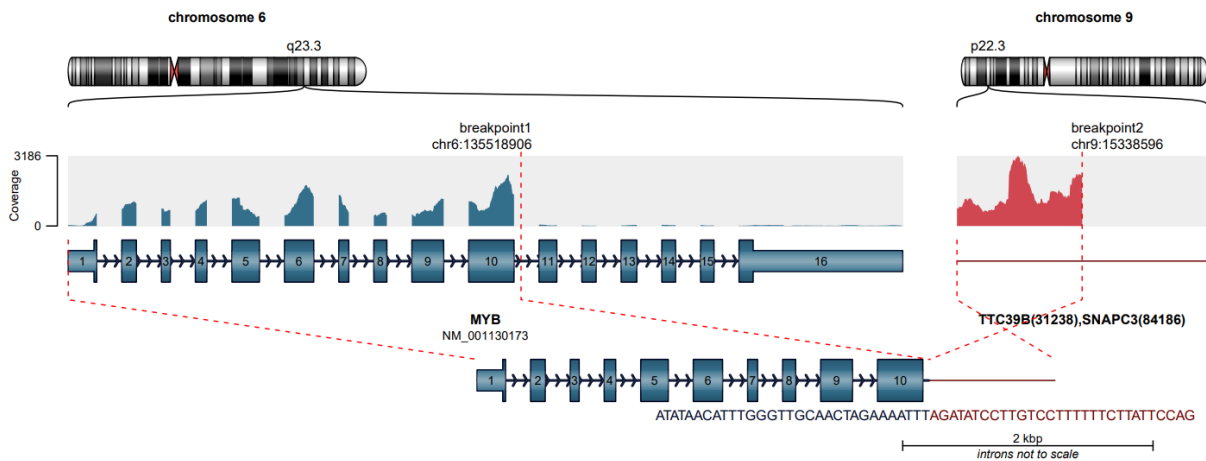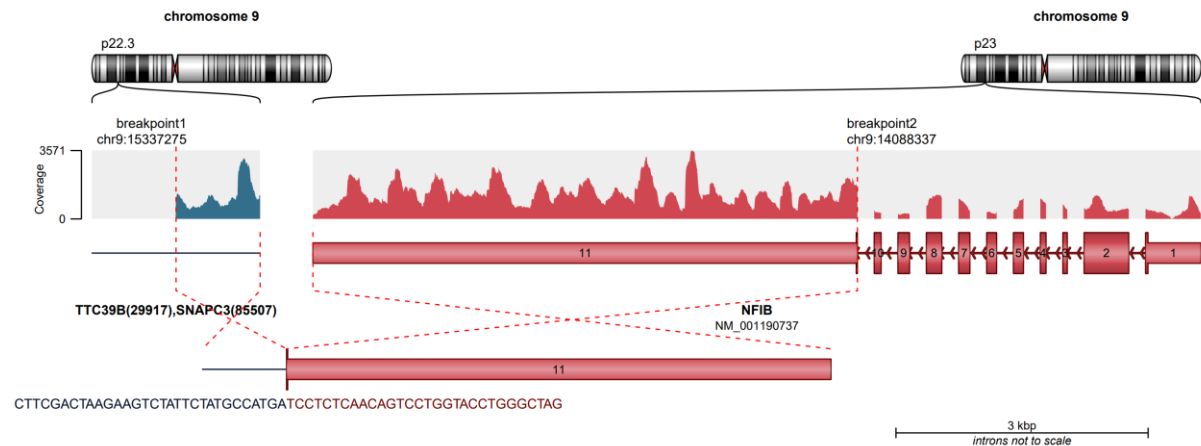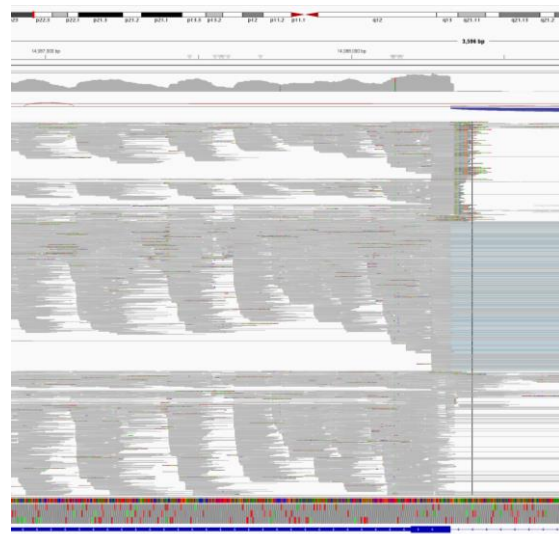

NFIB

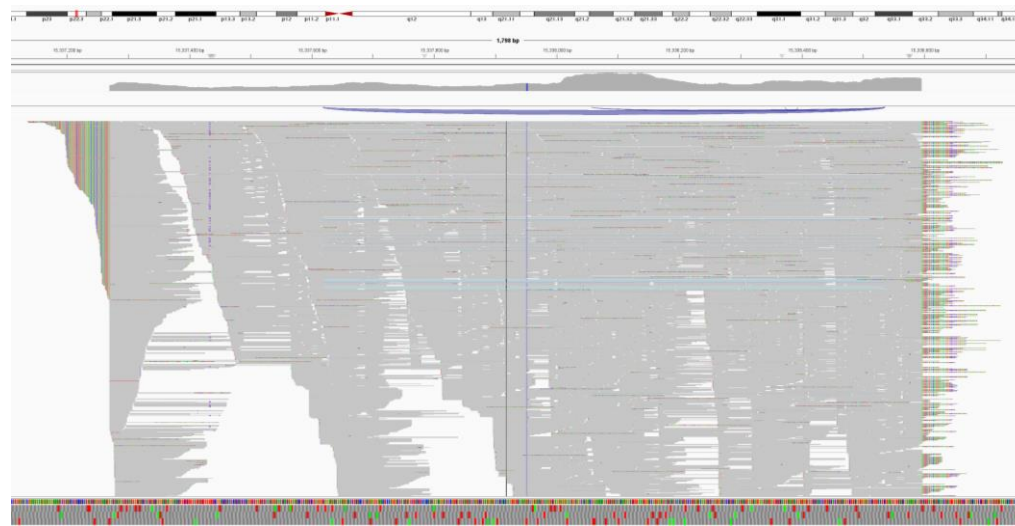

Intergenic 1.3 kb

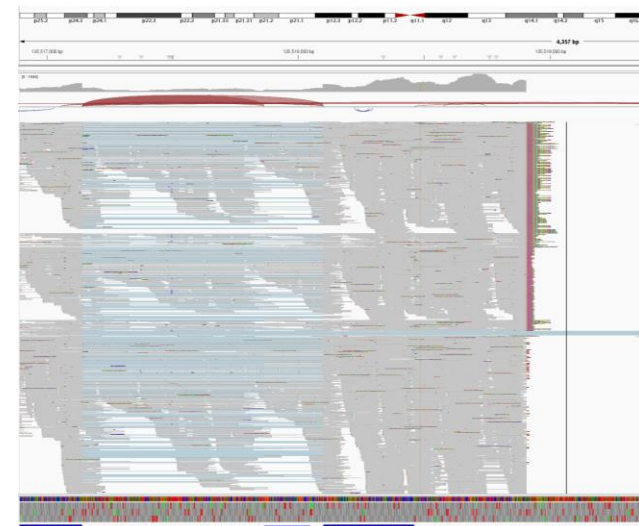

MYB

Supplement: Supplementary file 4 — Supplemental Figure 3 [file 41416_2025_3307_MOESM4_ESM.pdf]

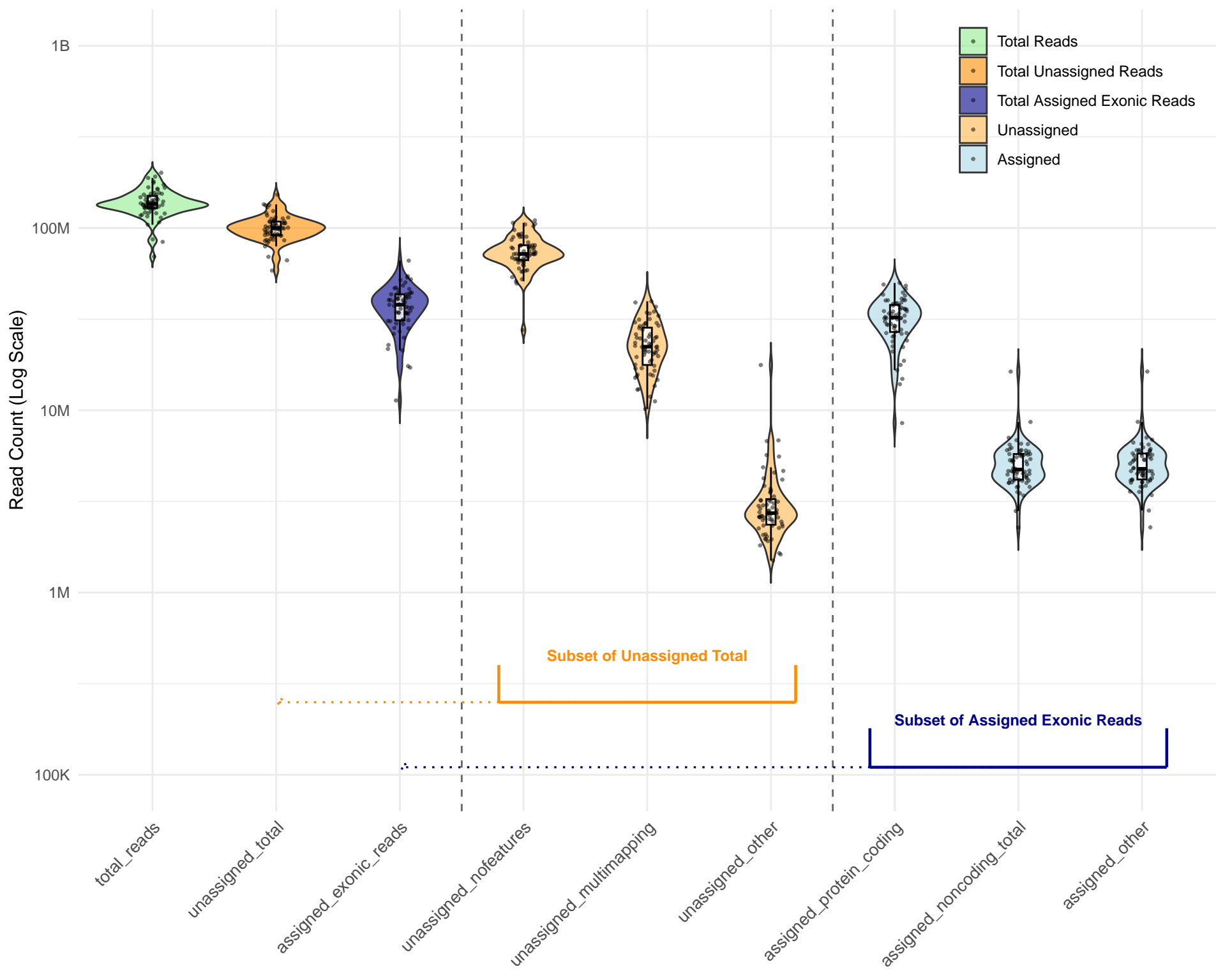

Supplement: Supplementary file 5 — Supplemental Figure 4 [file 41416_2025_3307_MOESM5_ESM.pdf]
